# Single-Cell and Population NF-κB Dynamic Responses Depend on Lipopolysaccharide Preparation

**DOI:** 10.1371/journal.pone.0053222

**Published:** 2013-01-03

**Authors:** Miriam V. Gutschow, Jacob J. Hughey, Nicholas A. Ruggero, Bryce T. Bajar, Sean D. Valle, Markus W. Covert

**Affiliations:** 1 Department of Bioengineering, Stanford University, Stanford, California, United States of America; 2 Department of Chemical Engineering, Stanford University, Stanford, California, United States of America; Ohio State University, United States of America

## Abstract

**Background:**

Lipopolysaccharide (LPS), found in the outer membrane of gram-negative bacteria, elicits a strong response from the transcription factor family Nuclear factor (NF)-κB via Toll-like receptor (TLR) 4. The cellular response to lipopolysaccharide varies depending on the source and preparation of the ligand, however. Our goal was to compare single-cell NF-κB dynamics across multiple sources and concentrations of LPS.

**Methodology/Principal Findings:**

Using live-cell fluorescence microscopy, we determined the NF-κB activation dynamics of hundreds of single cells expressing a p65-dsRed fusion protein. We used computational image analysis to measure the nuclear localization of the fusion protein in the cells over time. The concentration range spanned up to nine orders of magnitude for three *E. coli* LPS preparations. We find that the LPS preparations induce markedly different responses, even accounting for potency differences. We also find that the ability of soluble TNF receptor to affect NF-κB dynamics varies strikingly across the three preparations.

**Conclusions/Significance:**

Our work strongly suggests that the cellular response to LPS is highly sensitive to the source and preparation of the ligand. We therefore caution that conclusions drawn from experiments using one preparation may not be applicable to LPS in general.

## Introduction

The innate immune system has been evolutionarily conserved to exhibit distinct protective responses to a diverse set of microbial pathogens. The Toll-like receptor (TLR) family is primarily responsible for detecting the pathogen-associated molecular patterns (PAMPs) associated with viruses, bacteria, protozoa, or fungi. That information is relayed by the TLR family through cell signaling cascades that induce nuclear translocation of the Nuclear factor (NF)-κB family of transcription factors [1]. NF-κB controls or affects a diverse set of downstream processes including development, apoptosis, and coordination of the innate and adaptive immune systems, and consequently, aberrant NF-κB expression plays a role in the pathogenesis of several diseases, such as chronic inflammation and cancer [2], [3]. NF-κB activation has also been used extensively as a case study in the field of systems biology, which has led to a deeper understanding of system-level interactions. For instance, computational modeling of NF-κB, based on a combination of experimental and computational analyses, has expanded comprehension of the response to TNF-α [4]. Later studies have integrated computational models to elucidate responses to different ligands, additional signaling components, and single-cell behavior [5]–[9]. Detailed measurements of NF-κB activation dynamics have been facilitated by live-cell fluorescence microscopy, which has enabled researchers to visualize and measure how cellular population dynamics result from the accumulation of single cell responses [9]–[13]. Recently, the throughput of these approaches has been expanded, leading to novel insights regarding phenotypic heterogeneity within a population [14]. For example, it has been shown that single cells may show significant response variations when stimulated, even in a genetically identical cell population [15]. As another example, earlier population-level studies demonstrated a reduction in the amplitude of NF-κB activation as the concentration of tumor necrosis factor (TNF) was decreased [16]. Such amplitude reduction could be have two explanations: either (1) all cells exhibit similar response dynamics with uniformly reduced amplitudes; or (2) only a fraction of the cells respond, but the responding cells exhibit an equal amplitude across concentrations. High-throughput, single-cell microscopy experiments supported and quantified a combination of these two mechanisms [17]. Further examination of the NF-κB response has been conducted with the bacterial cell wall component, lipopolysaccharide (LPS). LPS, found in the outer membrane of gram-negative bacteria, induces NF-κB activation via binding to the TLR4 receptor [18]. Population-based measurements indicated that LPS causes sustained activation of NF-κB nuclear translocation for approximately 3 hours, due at least in part to paracrine signaling via secreted TNF [5]. Such translocation was also seen in single-cell studies; however, these experiments also showed at least one alternative response among a smaller yet significant population of cells [15]. In that population, NF-κB underwent only transient translocation, with some cells exhibiting oscillatory behavior. These results suggest that population-level responses can be an accumulation of qualitatively different single-cell behaviors, and that in some cases, correctly interpreting population-level experiments may be difficult.

The NF-κB response to LPS is now known to depend on the source and preparation of the ligand. For example, preparations that are insufficiently purified often contain bacterial lipoproteins, which can bind TLR2 to induce a response [19]. Furthermore, the shape of the lipid A component of LPS – conical versus cylindrical – can also effect receptor binding and subsequent response [20].

We therefore hypothesized that the population and single-cell NF-κB responses to LPS would vary according to the preparation, and set out to quantify these differences using our high-throughput pipeline. Here we describe the NF-κB response to three selected preparations, including one highly purified source as well as the commonly used Sigma-Aldrich source, across multiple concentrations and in the presence and absence of soluble TNF receptor.

## Results

To determine the differences in NF-κB activation at both the single-cell and population level, we began by measuring time courses of p65-dsRed nuclear localization in response to each of three preparations – Sigma #L4524 from *E. coli* 055:B5 (Sigma), Invitrogen LPS-EB from E. coli 0111:B4 (EB) and a more highly purified form of EB called Ultrapure (UP) – at a single concentration (0.5 µg/mL). The concentration was chosen because it is common to much of the published literature, as well as our own work [5], [15].

First, we seeded cells onto glass-coverslip 96-well plates. These cells express a p65-dsRed fusion protein, driven by the endogenous mouse p65 promoter, as well as H2B-GFP under control of the ubiquitin promoter (to facilitate cell segmentation and tracking). We then simultaneously stimulated each well with one of the described LPS preparations, on an incubated epifluorescent microscope. Upon stimulation, active p65 fusion proteins shuttle into the nucleus, leading to changes in nuclear fluorescent intensity. LPS response dynamics were measured by the change in nuclear fluorescent intensity over time, normalized by the cytoplasmic intensity prior to stimulation.

**Figure 1:**
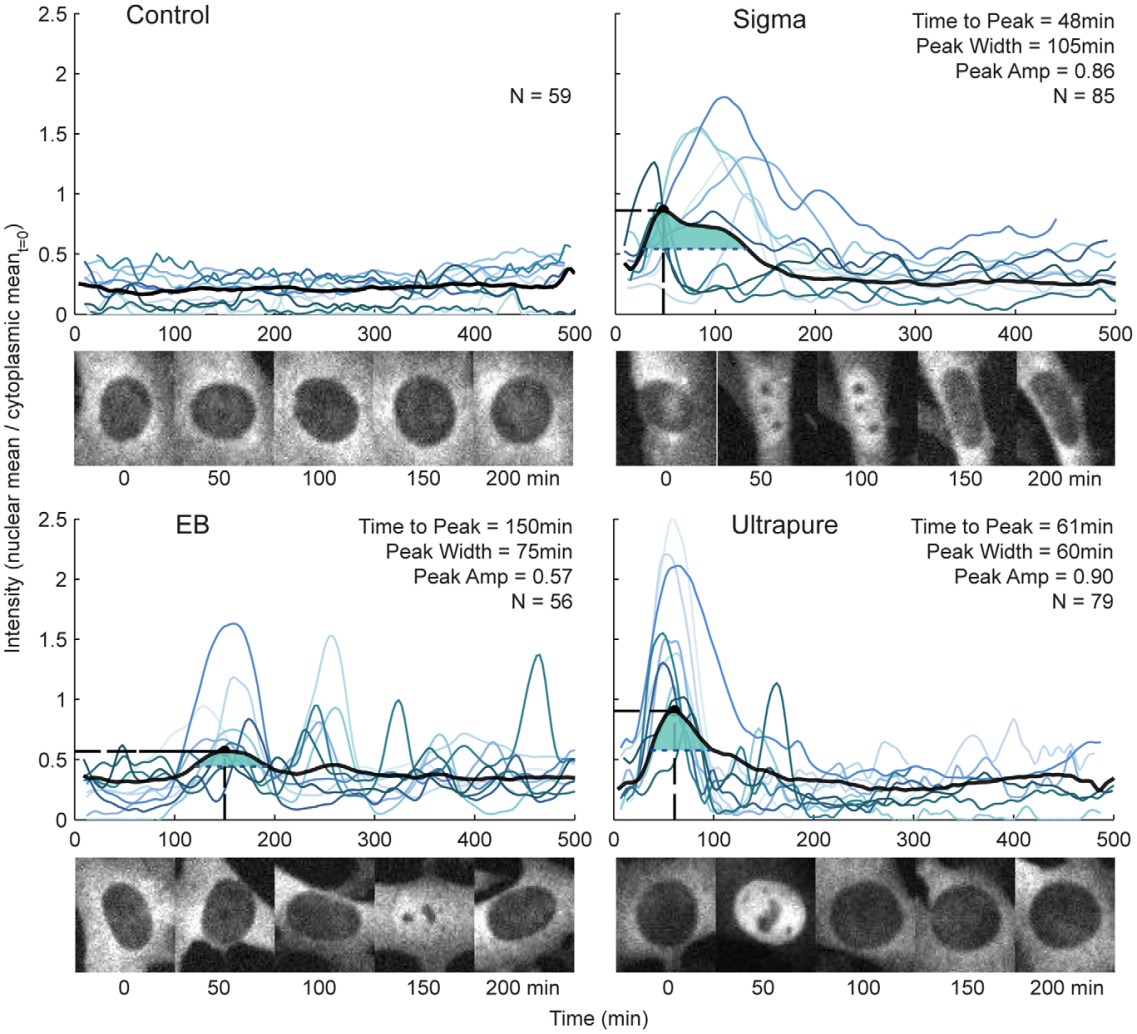
Comparison of single-cell NF-κB activation dynamics for three different LPS preparations. Intensity represents the relative nuclear localization of p65-dsRed fusion protein, calculated as mean nuclear intensity divided by initial mean cytoplasmic intensity. The concentration for all three preparations was 0.5 µg/mL. The black line shows the average time course for all cells; the light blue traces are ten randomly selected individual cells. The number of active cells (N), as well as the maximum peak amplitude (Peak Amp), and time elapsed until the maximum amplitude is reached (Time to Peak) are also shown. The maximum intensity is indicated by a dot and the two dashed lines indicate how Peak Amp and Time to Peak are determined. The duration of the first peak (Peak Width) is also shown. This value is determined by drawing a horizontal line at the intensity that is halfway between the minimum and maximum peak value. The region above the line and shaded in green denotes the time during which the p65-dsRed nuclear intensity is more than half of the maximum p65-dsRed nuclear intensity. Below each plot, corresponding representative microscope images are shown for the first 200 minutes after stimulation, as labeled.

We found that the different preparations led to significantly different activation dynamics (Figure 1). For example, cells stimulated with Sigma exhibited a 40–55% longer average localization than cells stimulated with EB or UP. We also noted that Sigma-stimulated cells had a marked qualitative heterogeneity in the response at the single-cell level, with both transiently- and persistently-activated cells, as we have previously described [15]. Cells stimulated with EB exhibited 51% lower-intensity activation and a three-fold longer time-to-peak on average, and were more likely to have subsequent strong secondary nuclear activation. The UP-stimulated cells had the highest peak intensity, about 17% higher than for Sigma, as well as a 45% longer time-to-peak.

A possible explanation of these observations is that the difference in activation is simply due to a potency effect – for example, a given concentration of EB is equivalent to a lower concentration of Sigma and UP. To test this hypothesis and further compare the three LPS preparations, we determined the range of LPS concentrations that activate cells for each preparation. We treated our cells with varying concentrations of Sigma, EB and UP, spanning nine orders of magnitude in concentration, and determined the nuclear localization over time for roughly 100 cells in at least three experiments at each concentration and preparation.

**Figure 2:**
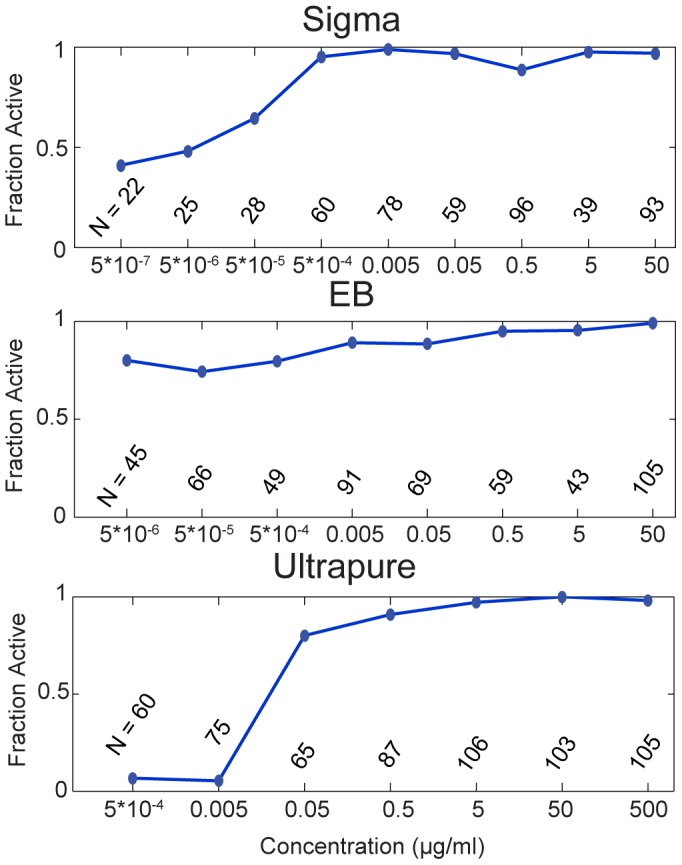
The potency window for each of the LPS preparations. The fraction of active cells is plotted as a function of concentration for values spanning nine orders of magnitude, as shown.

We first attempted to determine the potency range in terms of the fraction of cells that were visibly activated. Figure 2 shows the active cell fraction over the entire concentration range for each LPS preparation. We found that the resulting activation profiles were strikingly different for each preparation. Specifically, Sigma was maximally active beginning at concentrations of 5·10^−4^ to 5·10^−3^ µg/mL, while UP reached its peak fractional activation at 0.5–5 µg/mL, 2–3 orders of magnitude higher. UP LPS was also the only preparation that was essentially ineffective at the lowest concentrations we tested. Surprisingly, even though EB activation exhibited the lowest intensity in Figure 1, almost all of the EB-stimulated cells showed detectable NF-κB nuclear translocation, even at the lowest concentrations we used.

**Figure 3:**
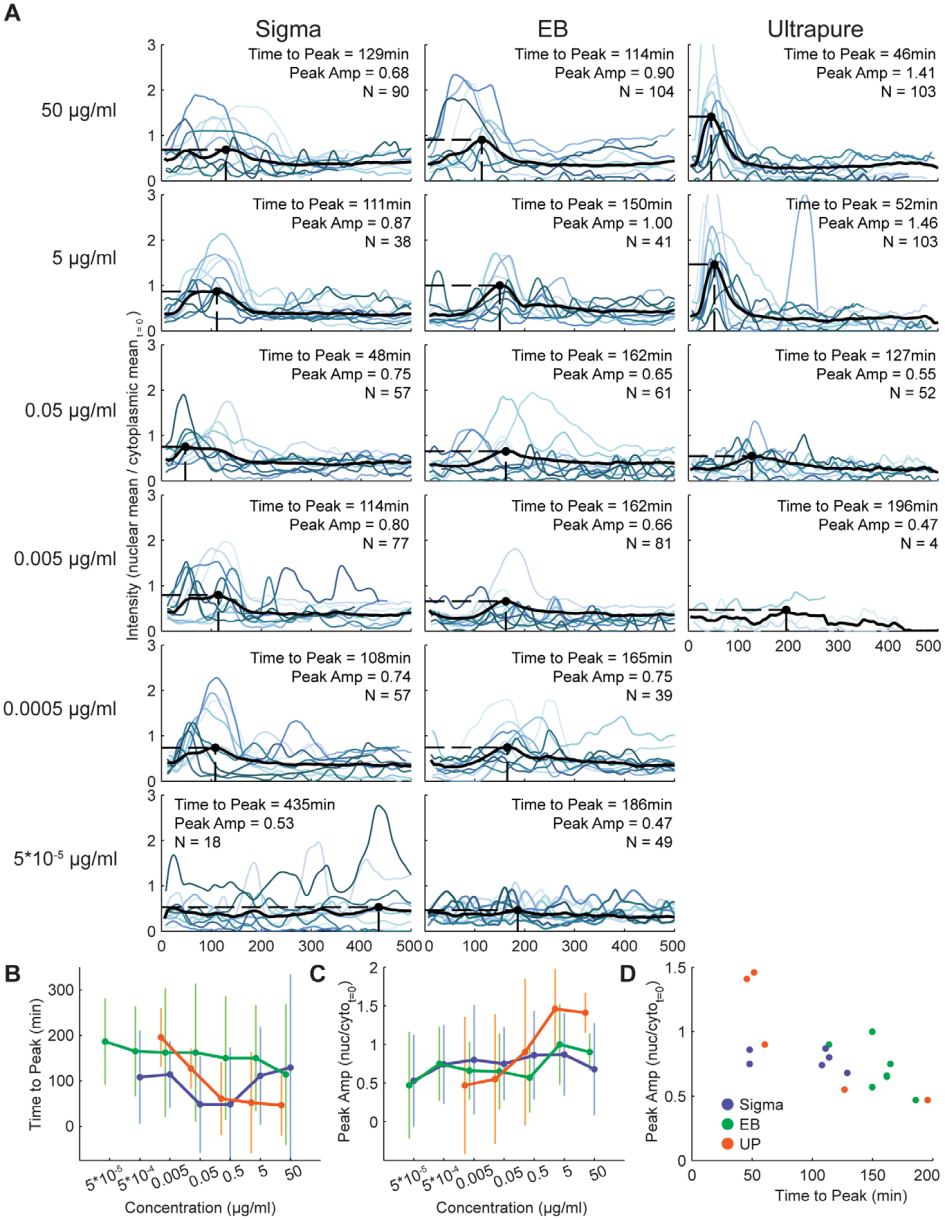
Activation dynamics for each of the LPS preparations and several concentrations and summary statistical comparisons. (A) As in Figure 1, the average and ten randomly selected traces of active cells are shown, as well as Time to Peak, Peak Amp and N. See Figure 1 legend for more details. Concentrations are indicated at left, and the preparation at top. The very lowest and highest concentrations are not shown here (but appear in Figure 4), because virtually no cells were found to be active in the first case, and the traces are essentially identical to their nearest neighbor in the second. (B) Time-to-peak values for each LPS preparation are shown with standard deviations, across each concentration. LPS preparations are indicated with different colors as labeled in D. (C) Peak amplitude values for each LPS preparation are shown with standard deviations across each concentration. (D) The correspondence between time-to-peak and peak amplitude is shown for each LPS preparation, including all concentrations.

Comparison of the dynamics of the LPS response for each preparation is complicated by this broad difference in the activity range. As a result, we compared the activation dynamics for each preparation at all concentrations. The average and selected single-cell time courses for cells stimulated with LPS at various preparations and concentrations are shown in Figure 3A. As before, we found that the time-to-peak was generally longest for EB-stimulated cells, and depended on concentration for both Sigma- and UP-stimulated cells (Figure 3B). Furthermore, the peak intensity was comparable for all treatments, with the exception of UP-stimulated cells, for which intensity increased with concentration (Figure 3C). We also found an inverse relationship between peak amplitude and time-to-peak for UP-stimulated cells, similar to cells treated with TNF [17]. In contrast, cells stimulated with Sigma generally maintained the same peak amplitude regardless of changes in time-to-peak (Figure 3D).

**Figure 4:**
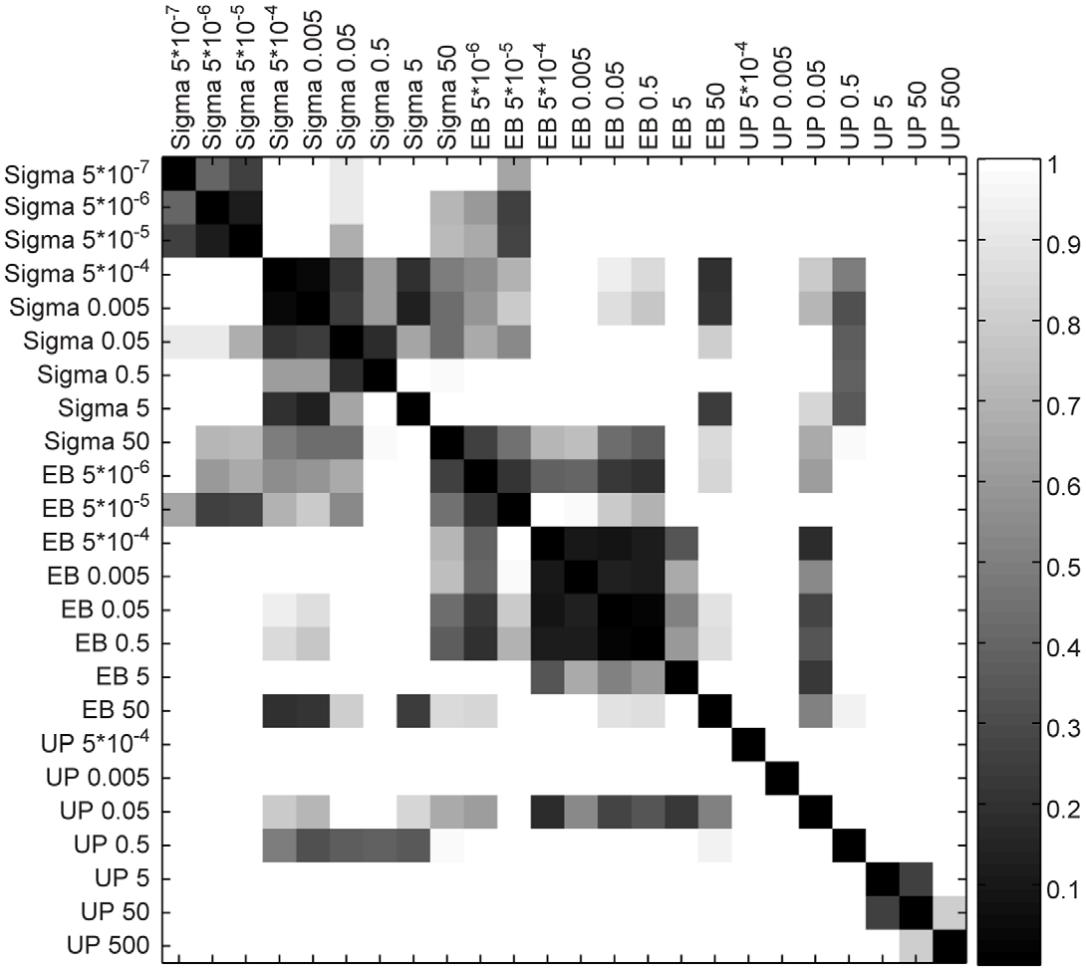
The similarity between average activation profiles across all concentrations and preparations. Similarity is calculated as cosine distance between pairs of average activation profiles. Pairs of time courses that are similar have a low vector distance; hence, the dark squares indicate similarity. The activation profiles are grouped by LPS preparation and sorted by concentration.

We then compared aggregate activation dynamics across all preparations and concentrations by determining the cosine distance between each pair of average time courses (Figure 4). The average time courses that are most similar have the shortest distance between their corresponding vectors. As expected, similarity between time courses often corresponds to cells treated with the same preparation of LPS and at similar concentrations. One interesting exception involves the UP preparation, for which the 0.05 µg/mL concentration produced an activation time course that was similar to many of the EB time courses. The similarity results from the sharp increase in average time-to-peak from the UP 5 µg/mL time courses to the UP 0.05 µg/mL time courses. The increase, from 52 minutes to 69 minutes to 129 minutes as concentration is decreased along this range, leads to a time-to-peak that is closer to the average EB time-to-peak across concentrations. With that exception, these findings do not support the hypothesis that the differences in NF-κB activation between preparations are related to a potency effect.

**Figure 5:**
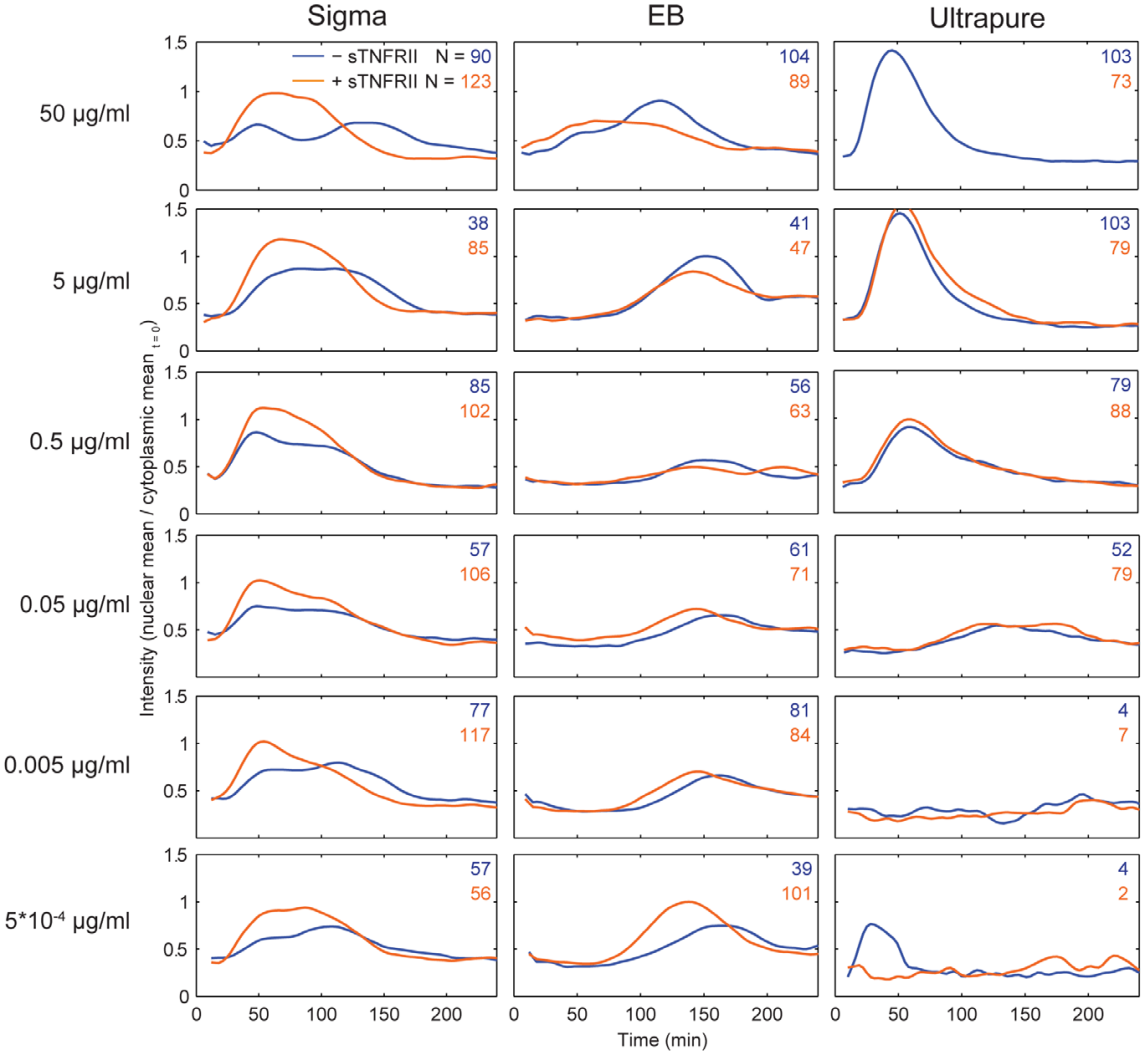
Blocking paracrine signaling by TNF across all concentrations and preparations. The average time course for N number of active cells is plotted for cells stimulated in the absence (blue trace, top value of n) and presence (orange trace, bottom value) of sTNFRII, which competes to bind TNF. Concentrations are indicated at left, and the preparation at top.

**Figure 6:**
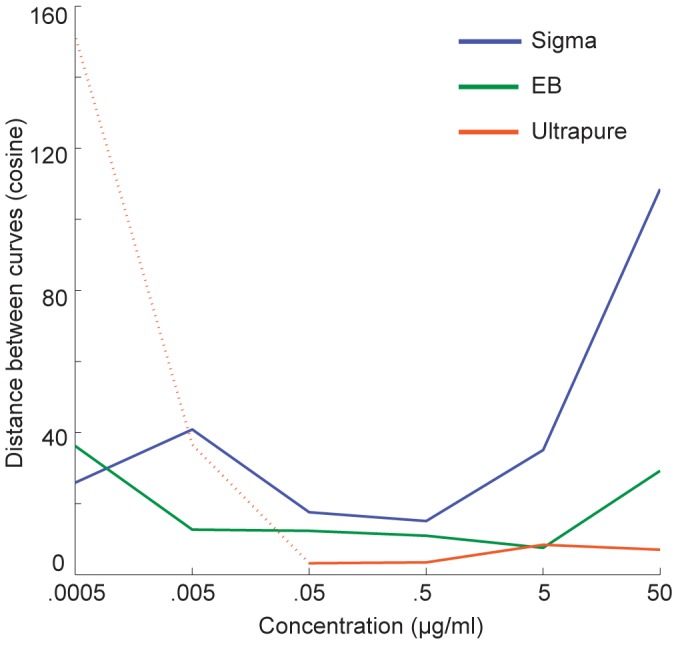
The effect of blocking TNF on the NF-κB activation time courses as a function of concentration and preparation. The cosine vector distances between the average time courses in the presence and absence of sTNFRII are shown. Almost no cells were activated by UP LPS at low concentrations; hence the dashed light blue line is included for completeness but is not statistically defensible.

Finally, we wondered how TNF secretion shaped the dynamics of NF-κB for each preparation of LPS. In our earlier work we found that cells treated with 0.5 µg/mL Sigma LPS had significantly different aggregate behavior when soluble TNF receptor II (sTNFRII) was added to the medium [5], [15]. In particular, the number of persistent cells was greatly reduced, suggesting that persistent activation depended on secreted TNF. We wondered if such sTNFRII sensitivity was a common feature of the LPS preparations. Accordingly, we treated cells simultaneously with sTNFRII and Sigma, EB or UP LPS over the same concentration range, and compared average time courses of NF-κB nuclear localization in the presence or absence of sTNFRII (Figure 5). We also calculated the cosine distance between the curves of average NF-κB intensity for active cells with or without sTNFRII (Figure 6). The responses of cells treated with high concentrations of Sigma were much more likely to be affected by sTNFRII, especially at early times (<100 minutes), than the responses of cells exposed to EB or UP (Figure 6). These results suggest that an important phenotype and commonly measured phenotype, namely secretion of a cytokine, can vary depending on the nature of the LPS preparation. We also performed high-sensitivity ELISA at multiple timepoints from 0 to 3 hours, but interestingly, detected no TNF in the media for any of the LPS preparations (<10 pg/mL, data not shown).

## Discussion

In summary, we found a significant dependence of the cellular NF-κB response to LPS on the preparation. This dependence is not simply a matter of concentration, as we showed by determining the potency window as well as by comparing the average response across concentrations and preparations. We also found that this difference could also lead to more extensive phenotypic differences, such as in the secretion of TNF.

Based on these findings, we believe that conclusions drawn from experiments using one specific preparation may not be applicable to every form of LPS. We further anticipate that, for studying the role of TLR4 in innate immune signaling, the UP preparation may be preferable as it is thought to act solely via TLR4, while the other preparations may engage other receptors. However, the Sigma preparation remains a valuable source of LPS because of the phenotypic heterogeneity that it can reliably generate in cells, particularly at higher concentrations. In any case, the LPS preparation used for any study needs to be carefully considered and duly noted, and in some cases, multiple preparations may be required to draw general conclusions.

Only three LPS preparations were considered here, and all come from an *E. coli* source. Hundreds of LPS variations exist in *E. coli* alone, not to mention the additional variation in preparation technique, and the cellular response to LPS has been shown to be extremely sensitive to certain molecular variations [21], [22]. For example, one study showed that adding a single phosphate group can change downstream pathway activation between the MyD88-associated and TRIF-associated pathways [23]. Moving forward, we note two observations for which we currently have no explanation: first, that the addition of sTNFRII actually appears to shorten the NF-κB time-to-peak; and second, that although Sigma LPS treatment leads to a significant increase in TNF gene expression [5], no soluble TNF can be detected in the culture medium by ELISA. Further studies are necessary to resolve these issues and thereby to better define the mechanism of LPS-induced TNF signaling.

Finally, it would be of great interest to characterize the complete phenotypic space of cellular NF-κB response to any and all types of LPS– for example, the LPS differences in pathogenic and non-pathogenic strains of *E. coli*. The LPS differences between broadly different strains of bacteria [20] are likely to also be reflected in the cellular NF-κB response, which could yield insight into whether the innate immune system is able to detect specific bacterial types or even strains.

## Materials and Methods

### LPS

We used three different LPS preparations. Sigma LPS (Sigma L4524, from *E. coli* 055:B5), the standard used in most LPS studies, is purified by ion-exchange chromatography, and is less than 1% protein and less than 1% RNA. As described by Sigma, it has been found to stimulate both TLR2 and TLR4. We also used Invivogen LPS-EB (Invivogen tlrl-eblps, from *E. coli* 0111:B4), which is extracted by phenol-water mixture. As described by Invivogen, it additionally contains lipopeptides, and stimulates TLR2 as well as TLR4. Lastly, we used Invivogen LPS-EB Ultrapure (Invivogen tlrl-pelps, also from *E. coli* O111:B4), which was extracted through successive hydrolysis steps and purified by phenol-TEA-DOC extraction. According to Invivogen, it activates only TLR4. The various dilutions of LPS in PBS (pH 7.4, Invitrogen) were kept on ice until just before stimulation, when warmed to ambient temperature. Soluble TNF Receptor II (R & D Systems, 426-R2) was used at 5 µg/ml for all concentrations of LPS.

### Cells

We used a strain of mouse relA^−/−^3T3 fibroblasts courtesy of the Baltimore Lab [24], that were infected with lentivirus to drive expression of two different transgenes, as described previously [15], [17]: p65-dsRed under control of the endogenous mouse p65 promoter, and H2B-GFP driven by the human ubiquitin C promoter. The p65-dsRed dynamics were phenotypically similar to the p65-GFP construct that we used previously [15].

### Cell Culture

Cells were cultured in DMEM (Invitrogen 11965-092) supplemented with 2 mM L-glutamine (Gibco 25030), 100 U/ml penicillin, 100 µg/ml streptomycin (Gibco 15140), and 10% fetal bovine serum (Omega Scientific, FB-11, lot 105247). We used T75 and T25 plasma-coated flasks for culture.

### Imaging

Approximately 20 hours prior to imaging, cells were seeded at about 7,000 cells/well onto glass coverslip 96-well imaging plates (Nunc, 164588) that had been pre-coated with 10 µg/ml fibronectin (Millipore FC010). An hour before imaging, media was changed to imaging media (DMEM prepared without phenol red, with 1% FBS). Dilutions of LPS (in PBS) were added to media, and left for the remainder of the experiment. Cells were imaged with a Nikon Eclipse Ti fluorescence microscope controlled by Micromanager, in both FITC and TRITC channels (Semrock), every 5–6 min for 120 frames. Temperature (37°C), CO_2_ (5%), and humidity were held constant during the experiments. Segmentation, cell tracking, and curation of the images was performed using custom Matlab software. Cells were manually defined as active, with the criteria that nuclei were uniformly brighter than at timepoints prior to stimulation, with visible nucleoli.
